# Proceedings of the 2025 society for Hematopathology/European association for Haematopathology workshop session 1: new entities in pediatric acute leukemia

**DOI:** 10.1007/s12308-026-00745-y

**Published:** 2026-11-12

**Authors:** David D. Grier, Michael J. Borowitz, Olga K. Weinberg, Alexandra E. Kovach

**Affiliations:** 1 Division of Pathology, Cincinnati Children’s Hospital Medical Center, Cincinnati, OH USA; 2 Department of Pathology, The Johns Hopkins Hospital, Baltimore, MD USA; 3 Department of Pathology, University of Texas Southwestern Medical Center, Dallas, TX USA; 4 Departments of Laboratory Medicine and Pathology, Boston Children’s Hospital, Boston, MA USA

**Keywords:** Acute leukemia, Pediatric, B-ALL, T-ALL, AML, Myeloid sarcoma, MPAL, *KMT2A*, *PICALM*, *SMARCB1*

## Abstract

Current classifications of acute leukemia now incorporate a large number of new entities defined by recently described unique genetic drivers. The workshop cases highlighted many of these subtypes and also argued for inclusion of additional entities in future classifications, including *KMT2A*-rearranged B-ALL/LBL with surface light chain expression, *PICALM*-rearranged leukemia/lymphoma, and *SMARCB1*-deficient hematopoietic malignancies.

## Introduction

The classification of acute leukemia has undergone a fundamental paradigm shift over the past decade, driven by the widespread adoption of large-scale genomic technologies – including next-generation sequencing (NGS), RNA sequencing (RNA-Seq), and optical genome mapping (OGM) – that have exposed the molecular heterogeneity underpinning these neoplasms. Gene expression profiling and comprehensive genomic studies have now largely defined the primary genetic drivers of B-lymphoblastic leukemia (B-ALL) [1–4], and both the World Health Organization 5th Edition (WHO 5th Ed.) and the International Consensus Classification (ICC) now organize B-ALL, T-lymphoblastic leukemia/lymphoma (T-ALL), and acute myeloid leukemia (AML) according to these primary genetic drivers, superseding earlier frameworks that relied predominantly on morphology and immunophenotype [5–7].

This reorganization has had direct and consequential implications for diagnostic pathology practice. Entities once grouped under broad “not otherwise specified” (NOS) categories are now resolvable into biologically and prognostically distinct subtypes, many carrying significant therapeutic implications, including eligibility for targeted therapies and risk-stratified treatment intensification. At the same time, provisional classifications in the ICC, including the multiple T-ALL transcription factor-driven subtypes [8–10] reflect a rapidly evolving taxonomy in which definitive classification often depends on methodologies not yet uniformly implemented across institutions.

Herein, we present the proceedings of a workshop session examining 56 submitted cases of acute leukemia, encompassing B-ALL, T-ALL, AML, myeloid sarcoma (MS), and mixed phenotype acute leukemia (MPAL). The cases were curated not only to highlight diagnostically instructive examples of newly defined and provisional genetic entities, but also to test the boundaries of current classification schemas. A number of illustrative themes emerged from the submitted cases. These included the expanding repertoire of laboratory methodologies capable of identifying these new genetic drivers. Relatedly, classification challenges arising from immunophenotypic ambiguity, lineage plasticity, and monocytic lineage switch, particularly in entities such as *DUX4*-rearranged B-ALL [11–14], *ZNF384* rearrangement, and *PAX5*P80R mutations, were addressed. In addition, special considerations governing B-ALL diagnosis in the context of constitutional trisomy 21 (Down syndrome) [15, 16], the phenomenon of surface immunoglobulin light chain expression in *KMT2A*-rearranged B-lymphoblastic lymphoma [17, 18]. and divergent classification outcomes between the WHO 5th Ed. and ICC in cases with co-occurring molecular alterations, such as concurrent *KMT2A* rearrangement and *TP53* mutation, were considered. Newly described AML subtypes, including AML with *UBTF*tandem duplication [19, 20], AML with *NPM1::MLF1*, and *PICALM*-rearranged leukemias [21–23], were similarly represented. Collectively, these cases underscore the imperative for integrated clinicopathologic and molecular classification and highlight entities warranting formal recognition in future iterations of international leukemia classification schemes [5–7].

## B-lymphoblastic leukemia (B-ALL)

B-ALL is the most common malignancy of childhood [24], and an overall profound success story in terms of improvements in clinical outcomes over time [25, 26]. Gene expression profiling and other large-scale genomic studies, many of which were performed by investigators at St. Jude Children’s Research Hospital, have now largely defined the primary genetic drivers of B-ALL [1–4]. As with other forms of acute leukemia, current classification schemas are now organized according to genetic subtype [5–7]. Dr. Michael Borowitz, Professor Emeritus at Johns Hopkins and former Director of Hematopathology, preceded the session with a lecture, “New Entities in Acute Lymphoblastic Leukemia” in which he focused on developments in genetic subclassification of B-ALL.

Table 1 depicts the -WHO 5th Ed. classification [5, 7] alongside the ICC [6] nomenclature for B-ALL and highlights the subtypes that were submitted to this workshop (*n* = 26). Several submitted cases highlighted interesting diagnostic aspects of existing genetic categories (cases #29, 49, 94, 150, 341, and 357). One discussed challenges of B-ALL diagnosis in the setting of paraneoplastic eosinophilia (#281). A unique case of B-lymphoblastic lymphoma with apparent *EWSR1* rearrangement was also received (#399). Other cases highlighting educational themes are also discussed in this section, including the heterogeneous methodologies used to identify novel genetic drivers, classification considerations in the setting of *DUX4* rearrangement and in Down syndrome, and a unique differential diagnosis with more typically adult-onset mature B-cell.

**Table 1:** WHO and ICC classifications of B-ALL and associated workshop cases

WHO 5th Ed	ICC	Workshop case(s) [Number: first author, institution]
*BCR::ABL1*	t(9;22)(q34.1;q11.2)/*BCR::ABL1*	
	with lymphoid only involvement	
	with multilineage involvement	150: Strong M, New York University Langone Health
*BCR::ABL1*-like	*BCR::ABL1*-like, ABL-1 class rearranged	
	*BCR::ABL1*-like, JAK-STAT activated	357: Broussard JB, Children’s Hospital Los Angeles 94: Hansen Smith MA, Children’s Hospital Los Angeles
	*BCR::ABL1*-like, NOS	
*ETV6::RUNX1*	t(12;21)(p13.2;q22.1)/ETV6::RUNX1	358: Asif H, Cleveland Clinic 101: Bauman SL, Cleveland Clinic
*ETV6::RUNX1*-like	Provisional: *ETV6::RUNX1*-like	452: Liu YC, St. Jude Children’s Research Hospital 5: Mariani RA, Phoenix Children’s Hospital
High hyperdiploidy	Hyperdiploid	29: Seth N, Long Island Jewish Medical Center
Hypodiploidy	Low hypodiploid	49: Wu SJ, Brigham and Women’s Hospital
	Near haploid	341: Youssef M, New York University Grossman School of Medicine
iAMP21	iAMP21	
*IGH::IL3*	t(5;14)(q31.1;q32.3)/*IL3*::IGH	
*KMT2A*	t(v;11q23.3)/KMT2A rearranged	205: Cook JR, Cleveland Clinic 376: Setiadi AF, British Columbia Children’s Hospital
*TCF3::HLF*	*HLF* rearrangement	201: Li J, Cincinnati Children’s Hospital
*TCF3::PBX1*	t(1;19)(q23.3;p13.3)/*TCF3::PBX1*	
Other	*DUX4* rearrangement	73: Banker, University of Missouri 327: Raca G, Children’s Hospital Los Angeles^a^
	*IKZF1* N159Y	500: Koo M, University of California Los Angeles
	*MEF2D* rearrangement	102: Wei Q, MD Anderson Cancer Center
	*MYC* rearrangement	480: Vijayanarayanan A, University of California San Francisco
	*NUTM1* rearrangement	
	*PAX5* P80R	269: Basha B, Seattle Children’s Hospital
	*UBTF::ATXN7L3/PAN3,CDX2 (“CDX2/UBTF”)*	
	*ZNF384*(*362*) rearrangement	
	Provisional: *KMT2A* rearranged-like	
	Provisional: *PAX5* alteration	270: Basha B, Seattle Children’s Hospital 192: Stalling M, Akron Children’s Hospital
	Provisional: *ZEB2* (p.H1038R)/IGH::CEBPE	
	Provisional: *ZNF384* rearranged-like	216: Wei Q, MD Anderson Cancer Center
NOS	NOS	158: Ali Hashim MM^b^, University Hospital, Columbia, Missouri^a^ 352: Vorapanya V, University of North Carolina Chapel Hill 281: Blain S, Haematologics 399: Sookdeo J, University of Tennessee Health Science Center

*ICC* International Consensus Classification [6], *NOS* Not otherwise specified, *WHO*World Health Organization, 5th Ed [5, 7]

^a^Oral presentation

^b^Presented by Banker L

### Laboratory methodologies for identification of novel genetic drivers

**Fig. 1:**
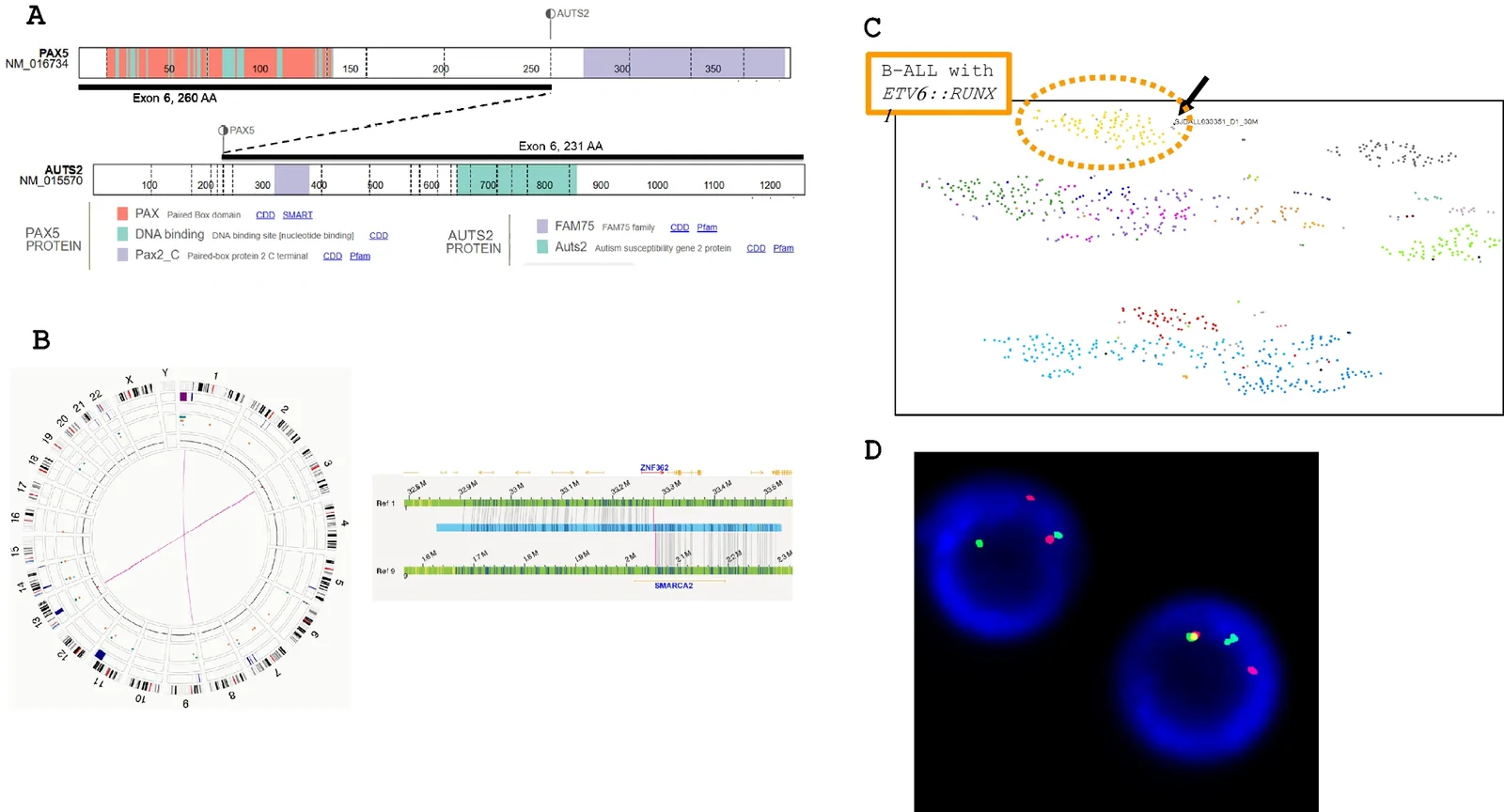
Some laboratory methodologies for identification of novel genetic drivers in B-lymphoblastic leukemia (B-ALL). **A** RNA sequencing data from case #192, B-ALL, PAX5alt, submitted by Dr. Melissa Stalling and colleagues, Akron Children’s Hospital, Akron, OH. **B** Optical genome mapping data from case #216, B-ALL, *ZNF384*-like, submitted by Dr. Qing Wei and colleagues, MD Anderson Cancer Center, Houston, TX. **C** Two-dimensional t-distributed stochastic neighbor embedding (t-SNE) plot from case #452, B-ALL, *ETV6::RUNX1*-like, submitted by Dr. Yen-Chun Liu and colleagues, St. Jude Children’s Research Hospital, Memphis, TN. The black arrow indicates the case. **D** Representative fluorescence in situ hybridization (FISH) image from case #5, B-ALL, *ETV6::RUNX1*-like, submitted by Dr. Rachel Mariani and colleagues, Phoenix Children’s Hospital, Phoenix, AZ

A majority of the submitted B-ALL cases (15/26, 58%) were characterized by unique translocations or point mutations. Many laboratories are using broad NGS panels as well as RNA-Seq. *PAX5* P80R (#269), *PAX*5alt [#192 (Fig. 1A), 270], and *IKZF1* (#500) mutations are readily identifiable by NGS. Examples of RNA-Seq detecting *MEF2D* rearrangement (#184) as well as a rearrangement consistent with *PAX5*alt (*PAX5::AUTS2*; #192) were also submitted. Clinical utility of specific fusion breakpoint identification by OGM was demonstrated in a case of *MEF2D* rearrangement (#184) and also on a B-ALL, *ZNF384*-like case (*ZNF362::SMARCA2*, #216, Fig. 1B), a category which is provisional in the ICC [6] and unlikely to be detected by targeted fusion panels or other standard assays. Further benefits and challenges of OGM were detailed in this session in an oral presentation of T-lymphoblastic leukemia with *TLX3::BCL11B*, #454.

Similar to the *ZNF384*-like category, *ETV6::RUNX1*-like is provisional in the ICC [6] and related to its namesake *ETV6::RUNX1*through a common gene expression profiling signature [27]. Two cases each of *ETV6::RUNX1* (#101, 358) and *ETV6*::RUNX1-like (#5, 452) were received. *ETV6::RUNX1*is the most common genetic driver of B-ALL in childhood, accounting for approximately 25% of pediatric B-ALL and associated with an excellent prognosis even prior to the most modern therapies [28]. The two submitted cases with *ETV6::RUNX1* were detected by fluorescence in situ hybridization (FISH) probes on a standard B-ALL panel. The two *ETV6*::*RUNX1*-like examples highlight the breadth of testing modalities capable of identifying this recently described set of fusions [27]. One, submitted by Dr. Liu and colleagues from St. Jude Children’s Research Hospital, where whole exome, genome and transcriptome sequencing are clinical standard of care, also demonstrated clustering of the case with a cohort of *ETV6::RUNX1*-rearranged B-ALL by t-SNE plot (Fig. 1C). By contrast, given the presence of an *ETV6* rearrangement and non-*RUNX1* partner on standard B-ALL FISH panel, within the background of a CK with 12p13 (*ETV6* locus) rearrangement, an *ETV6*::*RUNX1*-like case was suspected (Fig. 1D). Another example of inference from classical cytogenetics of an important primary genetic driver was *HLF* rearrangement, an especially poor prognostic subtype of childhood B-ALL now recognized in the WHO [5] and ICC [6], from evidence of *TCF3* rearrangement without the more common *PBX1* partner by B-ALL FISH (#201). These latter cases emphasize the ability of widely available and cost-effective assays to identify at least some novel B-ALL genetic subtypes.

### Considerations regarding DUX4 rearrangement

**Fig. 2:**
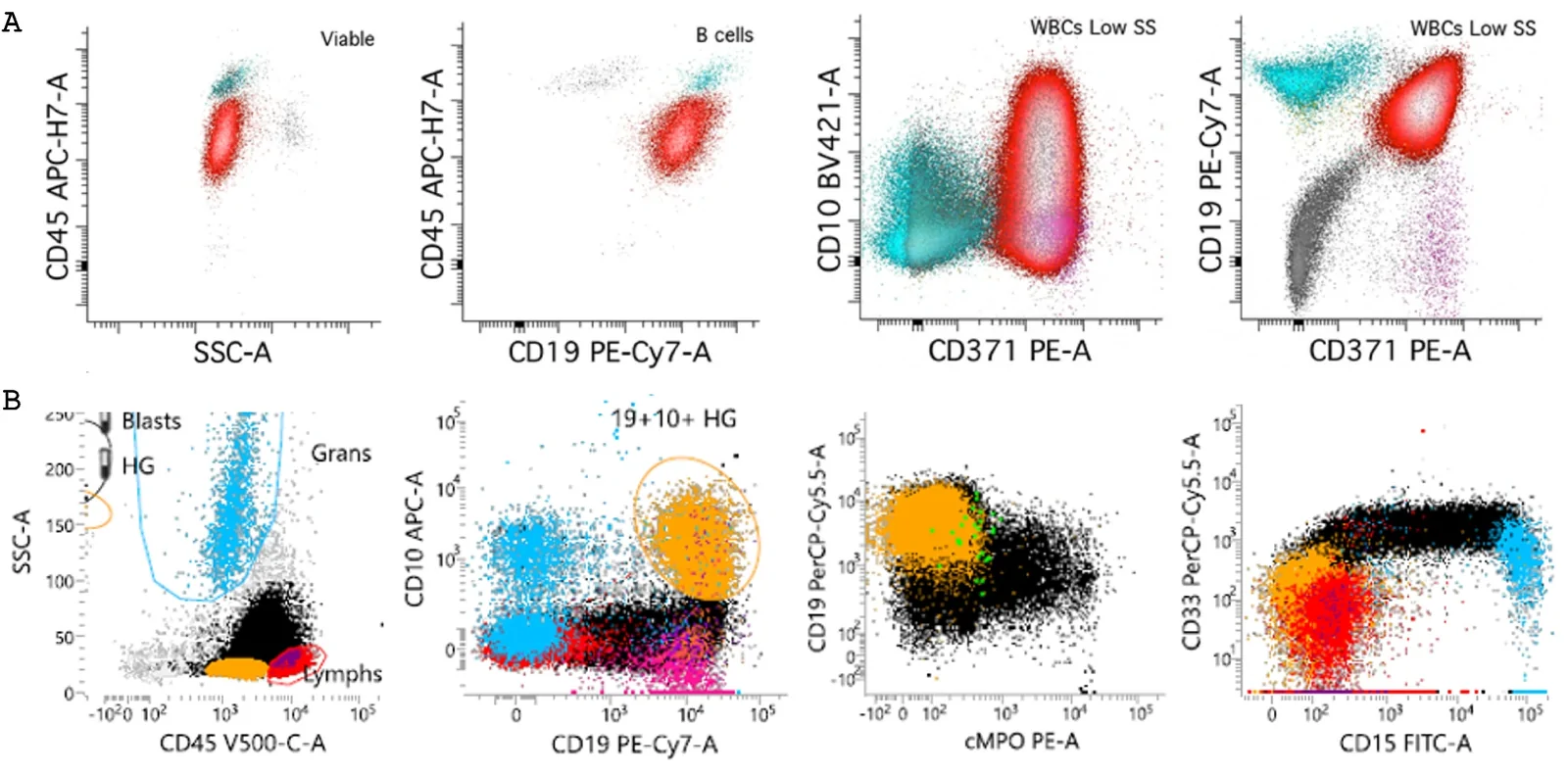
Flow cytometric features of B-ALL with *DUX4* rearrangement at diagnosis. **A** Top row, case #327, peripheral blood. Red, abnormal progenitor (blast) population; light blue, mature B cells; magenta, monocytes. Images courtesy of submitted workshop slides, Dr. Gordana Raca and colleagues, Children’s Hospital Los Angeles, Los Angeles, CA. **B** Bottom row, case #73, bone marrow aspirate. Orange, CD19-positive/CD10-positive component of abnormal progenitor (blast) population; black, CD19-variable/CD10-negative component of the blast population; red, mature lymphocytes; light blue, granulocytes. Images courtesy of submitted workshop slides, Dr. Linnea Banker and colleagues, University of Missouri, Columbia, MO

Initially described alongside *ETV6::RUNX1*-like [27] and *PAX5*alt [29], *DUX4*-rearranged B-ALL [30] can be particularly challenging to diagnose [11] as highlighted by the two cases submitted for this session (#73, 327). First, the prognosis is excellent such that it is important to identify, but it is cytogenetically cryptic, and secondary *ERG* deletions, present in a majority of cases, are often the only evidence to suggest a *DUX4*rearrangement [31]. A pathognomonic and readily detectable feature of this entity, though, is surface expression of CD371 (CLL-1) [32], illustrated in an oral presentation (#327; Fig. 2A). Although this case atypically lacked an *ERG*deletion [11, 12], the gene expression profile of *DUX4-*rearranged B-ALL was confirmed by transcriptome sequencing.

*DUX4-*rearranged B-ALL also frequently demonstrates evolving monocytic differentiation following induction chemotherapy; this is regularly observed in Europe, where day 15 post-induction bone marrow evaluations are routine [13]. When the monocytic differentiation is present at diagnosis, it raises the differential diagnosis of a less favorable prognosis leukemia, mixed phenotype acute leukemia (MPAL) B/Myeloid, also at times seen in *ZNF384* rearrangement and *PAX5*P80R [14]. Indeed, case #73 showed striking monocytic differentiation in the setting of a B-ALL “backbone” and an *IGH::DUX4* rearrangement (Fig. 2B). It is recommended that these cases be treated as B-ALL as the patients do very well, but certainly MRD analysis – by both flow and NGS – may be complicated by the fusion being harbored in the monocytic component as well as the B-cell component [12]. For these reasons, the panel diagnosis in this case was “B-ALL with *DUX4* rearrangement and monocytic differentiation.” This scenario highlights the likely widespread lineage plasticity of many acute leukemias, some of which are more evident than others, but further emphasizes the importance of integrated genetic rather than solely immunophenotypic classification. Diagnosis in this case was further confounded by a concurrent *IGH::RIN3* rearrangement by NGS, whose significance is uncertain.

### Classification of CRLF2 rearrangements in the setting of Down syndrome

Two cases (#158, 35) of B-ALL in patients with constitutional trisomy 21 (Down syndrome), were submitted as *BCR::ABL1*-like (Ph-like) due to *CRLF2* rearrangements. The panel diagnoses were “B-ALL not otherwise specified in the setting of Down syndrome.” Indeed, *CRLF2* rearrangement is common in B-ALL in Down syndrome patients, but recent large-scale data demonstrate that these fusions are most often not associated with a *BCR::ABL1*-like genetic expression profile [15] and that in the absence of documented *BCR::ABL1*-like genetic features, the Ph-like designation should be withheld. Although those Down syndrome patients with both *CRLF2*rearrangement and a Ph-like signature may have a worse prognosis, there are limited data on this point. Because Down syndrome patients are highly prone to chemotherapy-induced cytotoxicity, it has been recommended that this diagnosis be limited to non-Down syndrome patients [16].

### KMT2A-rearranged B-lymphoblastic leukemia with surface light chain expression: differential diagnosis with mature B-cell lymphoma

**Fig. 3:**
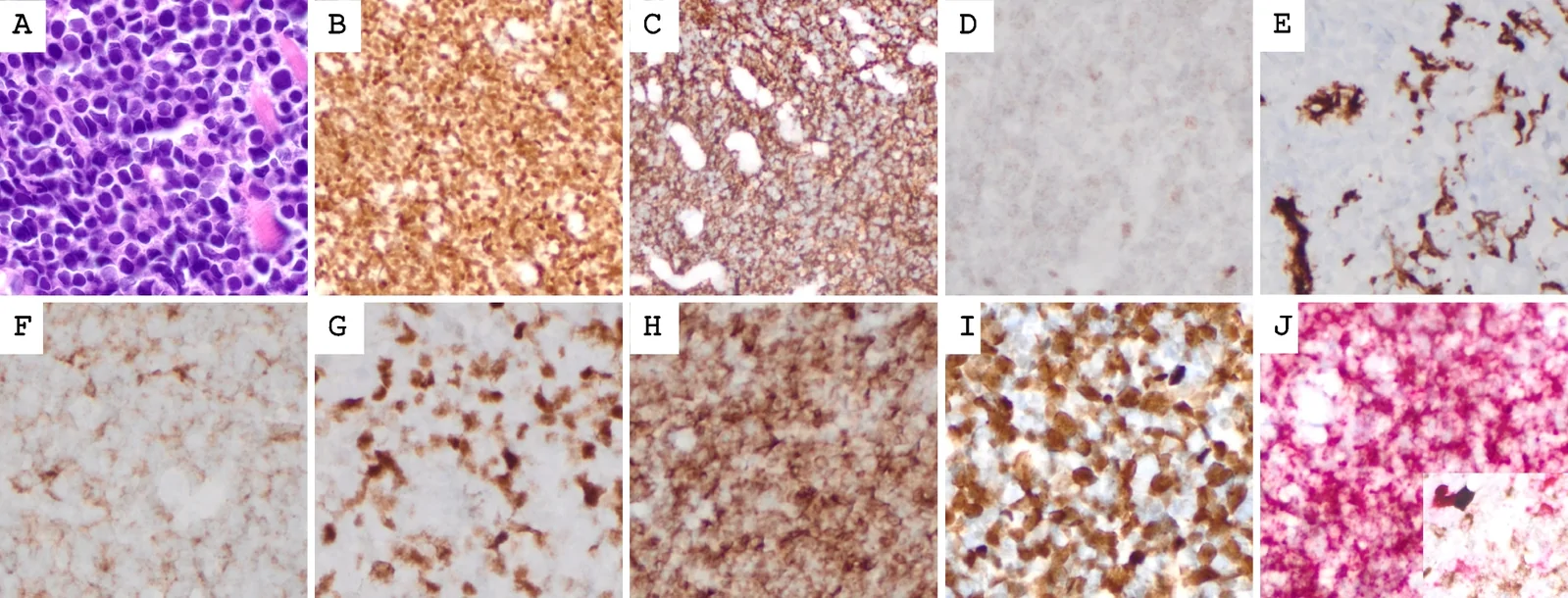
B-lymphoblastic lymphoma with *KMT2A* rearrangement and mature immunophenotype. Case #205, eyelid mass in an 11-month-old infant. **A** Hematoxylin and eosin (H&E). **B** PAX5; **C** CD20; **D** TdT; **E** CD34; **F** CD10; **G** C-MYC; **H** BCL2; **I** Ki67; **J** Ultrasensitive in situ hybridization stain for kappa (brown) and lambda (red); inset: tonsil control. Images courtesy of submitted workshop slides, Dr. James R. Cook and colleagues, Cleveland Clinic, Cleveland, OH

Two strikingly similar cases of *KMT2A*-rearranged B-lymphoblastic lymphoma presenting in the eyelid of young children were confounded in their diagnostic workups by surface immunoglobulin light chain expression (#205, 376). In #205 (Fig. 3), a *KMT2A::MLLT3* fusion was detected on involved bone marrow and subsequently confirmed by NGS of the eyelid mass. In #376, the immunophenotype was similar, with negative TdT and CD34, and lambda light-chain restriction. Break-apart FISH of the eyelid mass detected a *KMT2A* rearrangement; bone marrow was negative. Similar cases have previously been reported in one large case series [17] and two individual case reports [18]. Together with these workshop cases, the data credibly suggest calling out this phenomenon as its own entity in future classifications.

## T-lymphoblastic leukemia/lymphoma

T-lymphoblastic leukemia/lymphoma (T-ALL) is a neoplasm of T-lymphoblasts that accounts for 15% of all childhood lymphoblastic leukemia cases and 85–90% of all lymphoblastic lymphomas. The ICC has expanded to include several provisional T-ALL subtypes (Table 2), and the list of recognized genetic drivers continues to grow [8–10]. Eight new entities are classified based on aberrant activation of different families of transcription factors [6]. While many of these abnormalities can be detected by karyotyping, FISH, or PCR; comprehensive genomic analysis may be required. Four T-ALL cases were submitted to the workshop. Each had unusual cytogenetic or molecular findings, some cryptic by commonly used ancillary testing.

**Table 2:** WHO and ICC classifications of B-ALL and associated workshop cases

WHO 5th Ed	ICC	Workshop case(s) [Number: first author, institution]
ETP	ETP with *BCL11B* rearrangement	
	ETP NOS	
NOS	NOS	15: Brennan, Wake Forest 147: You-Wen, Roswell Park
	Provisional: *HOXA* dysregulated	149: Strong, NYU
	Provisional: *SPI1*-r	
	Provisional: *TLX1*-r	
	Provisional: *TLX3*-r	454: Alfattal, MD Anderson^a^
	Provisional: *NKX2*-r	
	Provisional: *TAL1-2*-r	
	Provisional: *LMO1-2*-r	
	Provisional: *BHLH*, other	

*ETP* Early T-precursor, *ICC* International Consensus Classification [6], *NOS* Not otherwise specified, *WHO*World Health Organization, 5th Ed [5, 7].; -r, rearrangement

^a^Oral presentation

An example of the ICC provisional entity *HOXA*-dysregulated T-lymphoblastic leukemia/lymphoma with *KMT2A* rearrangement (#149), occurred in a patient with enlarged lymph nodes but mild constitutional symptoms. Peripheral blood findings initially resembled autoimmune lymphoproliferative syndrome due to increased double-negative T-cells, but lymph node biopsy confirmed T-ALL.

Case #454 highlights a unique form of T-ALL characterized by a *TLX3*::*BCL11B* rearrangement, an example of the provisional entity *TLX3*-activated T-ALL. The immunophenotype was not early T-cell precursor (ETP)-ALL: CD1a+, CD8 + without HLA-DR, CD117 or myeloid antigen expression. The translocation does not produce a fusion protein; instead, it brings *TLX3* under the control of the *BCL11B* enhancer, leading to ectopic overexpression of TLX3. Its cryptic genomic nature explains why traditional cytogenetic and molecular techniques often fail to detect it, underscoring the diagnostic challenges. In this case, OGM revealed alterations that were cryptic by other methods. *BCL11B* alterations more often occur in acute leukemias with a variety of stem cell-like, ETP, T-ALL, and MPAL T/Myeloid or lineage-ambiguous immunophenotypes, and result in activation of *BCL11B* and not *TLX3* [33–35].

## Acute myeloid leukemia and myeloid sarcoma (MS)

Acute myeloid leukemia (AML) is a heterogeneous group of myeloid neoplasms defined by morphology, immunophenotype, and molecular/cytogenetic findings. AML is the second most common type of leukemia in children [36]. The most recent classifications (WHO 5th Ed. and ICC) have introduced systems of categorizing AML with increased emphasis on molecular and genetic findings over immunophenotype and morphology [5, 37]. MS is a well-defined mass of leukemic myeloid blasts outside the marrow. Infiltration of leukemic myeloid blasts without mass formation should not be considered MS. Table 3 shows the current WHO 5th Ed. and ICC of AML.

**Table 3:** WHO and ICC classifications of B-ALL and associated workshop cases

WHO 5th Ed	ICC
Acute promyelocytic leukemia with *PML*::*RARA* fusion	Acute promyelocytic leukemia (APL) with t(15;17)(q24.1;q21.2)/*PML*::*RARA*
	APL with other *RARA* rearrangements
AML with *RUNX1*::*RUNX1T1* fusion	AML with t(8;21)(q22;q22.1)/*RUNX1*::*RUNX1T1*
AML with *CBFB*::*MYH11* fusion	AML with inv(16)(p13.1q22) or t(16;16)(p13.1;q22)/*CBFB*::*MYH11*
AML with *KMT2A* rearrangements	AML with t(9;11)(p21.3;q23.3)/*MLLT3*::*KMT2A*
	AML with other *KMT2A* rearrangements
AML with *DEK*::*NUP214* fusion	AML with t(6;9)(p22.3;q34.1)/*DEK*::*NUP214*
AML with *MECOM* rearrangements	AML with inv(3)(q21.3q26.2) or t(3;3)(q21.3;q26.2)/*GATA2*; *MECOM*(*EVI1*)
	AML with other *MECOM* rearrangements
AML with other defined genetic alterations (rare fusions)	AML with other rare recurring translocations
AML with *BCR*::*ABL1* fusion	AML with t(9;22)(q34.1;q11.2)/*BCR*::*ABL1*
AML with *NPM1* mutation	AML with mutated *NPM1*
AML with *CEBPA* mutation	AML with in-frame bZIP *CEBPA* mutations
	AML and MDS/AML with mutated *TP53*^a^
AML, myelodysplasia-related	AML and MDS/AML with myelodysplasia-related gene mutations^a^
AML, myelodysplasia-related	AML with myelodysplasia-related cytogenetic abnormalities
AML, defined by differentiation	AML not otherwise specified (NOS)^a^
Myeloid sarcoma	Myeloid sarcoma

*ICC* International Consensus Classification [6], *NOS* Not otherwise specified, *WHO*World Health Organization, 5th Ed [5, 7]

^a^10–19% blasts: MDS/AML; ≥ 20% blasts: AML

**Fig. 4:**
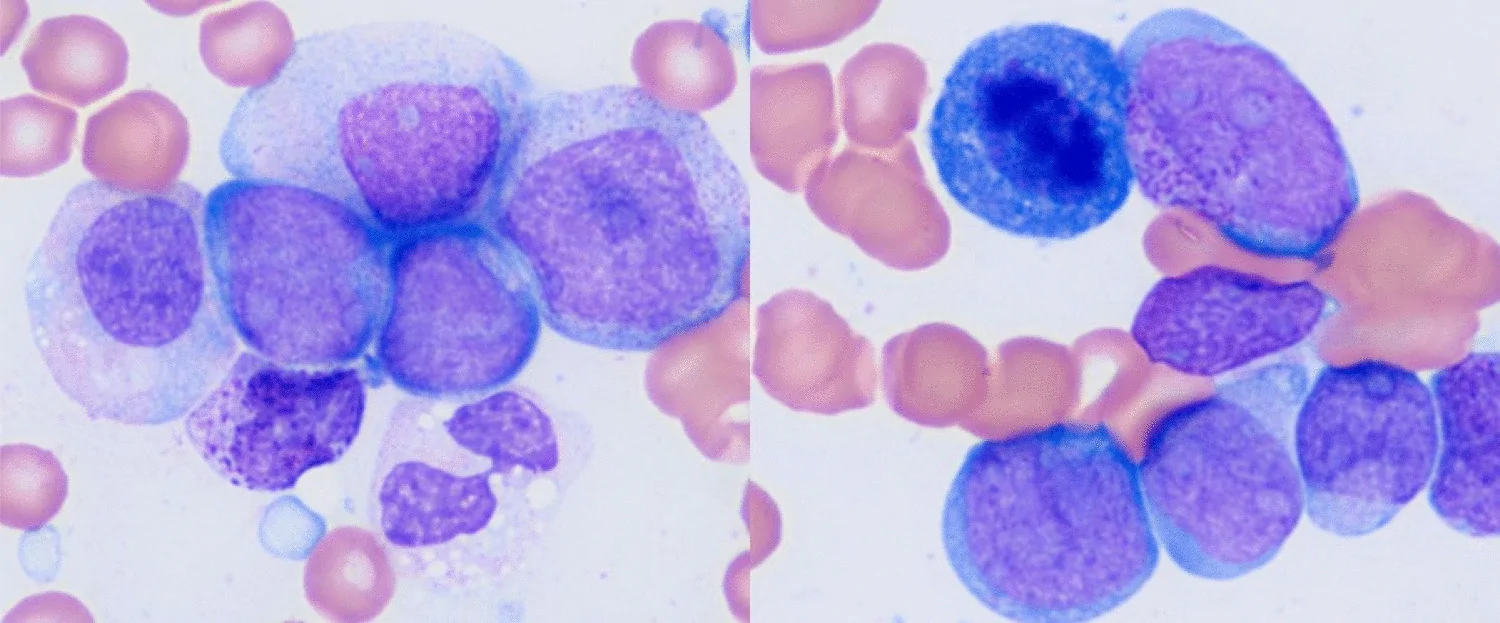
Acute myeloid leukemia with *DEK*::*NUP214* fusion with dysplastic myeloid and erythroid precursors. Case #139, bone marrow aspirate in 25-year-old female. Wright-Giemsa. Images courtesy of submitted workshop slides, Giby V. George and colleagues, University of Rochester Medical Center, Rochester, NY

Twenty-two cases of AML and MS were submitted to this session (AML patients with *NUP98* and infants with *KMT2A* rearrangements are covered in the Session 2 article on infant AML). Most cases fit into categories defined by the WHO 5th Ed. and ICC (Table 4). Among these, two cases (#25, #139) had *DEK*::*NUP214* fusions, which highlight the presence of dysplasia in non-MDS AML cases (Fig. 4). AML with *DEK*::*NUP214* fusion is a rare entity, accounting for less than 1% of pediatric AML cases and is generally considered aggressive. This leukemia subtype often presents with co‑occurring FLT3‑ITD mutations. MDS-related AML is rare in children, so that the presence of dysplasia reminds pathologists to consider other AMLs that can have morphologic dysplasia (especially *RUNX1*::*RUNX1T1* fusion, *DEK*::*NUP214* fusion, *NPM1*::*MLF1* fusion, *MECOM* rearrangements, AML with *NPM1* mutation). There were other established entities that had unusual morphologic or clinical presentations (#189 with APL-like clinical presentation, and #422 as an APL MS).

**Table 4:** Submitted cases of acute myeloid leukemia and myeloid sarcoma with recognized genetic driver

Case Number: First Author, Institution	Age (years)/Sex	Proposed Diagnosis	Panel Diagnosis	Comments
422: Guido, University of Miami	18/F	APL presenting as an isolated myeloid sarcoma	Agree	Isolated APL myeloid sarcoma
189: Wanes, MD Anderson	7/M	AML with *CBFB*::*MYH11*	Agree	Clinical and morphologic presentation mimicking APL
25: N. Lopez-Hisijos, Vanderbilt	18/M	AML with *DEK*::*NUP214*		Prominent morphologic dyserythropoiesis Fusion identified by karyotype
139: Giby, University of Rochester	25/F	*AML* with* DEK::NUP214*	Agree	Bone marrow and skin involvement Prominent multilineage dysplasia
52: Ondrejka Cleveland Clinic	16/M	Myeloid sarcoma/AML with *PICALM*::*MLLT10*	Agree	Mediastinal mass Diagnosis by IHC and NGS No bone marrow biopsy until 1 month post therapy
429: Petrova-Drus, Memorial Sloan Kettering	4/M	AML with *PICALM*::*MLLT10* fusion	Agree	Megakaryoblastic/erythroblastic differentiation
27: Stalling, Akron Children’s	13/F	Acute basophilic leukemia with *ETV6*::*HCK*	AML with *ETV6:*:*HCK* fusion	Previously unreported *ETV6::HCK* fusion
378: Younes, University of Minnesota	17/M	AML with *NPM1*::*MLF1*	Agree	New entity, previously classified as AML-MDS
242: Bell, Cleveland Clinic	10 months/F	Myeloid sarcoma/AML	Agree	Germline partial trisomy 18
266: Thuraisingam, University of Arizona	17/F	Evolving AML NOS	Agree	Evolving AML in a patient with Down syndrome No *GATA1* mutation
393: Kodgule Washington University^a^	18/M	AML NOS with leukemia cutis	Agree	Tandem duplications involving *UBTF* exon 13^a^

*AML* Acute myeloid leukemia, *APL* Acute promyelocytic leukemia, *IHC* Immunohistochemistry, *NGS* Next-generative sequencing, *NOS* Not otherwise specified

^a﻿^Oral presentation

There were several cases that fell under either or both the ICC designation of “AML with other rare recurring translocation” or the WHO 5th Ed. “AML with other defined genetic alterations” categories. These AML entities are rare and may be difficult to diagnose with routine AML FISH panels. There were two cases of AML with *PICALM*::*MLLT10* (#52, #429). #52 was challenging from a diagnostic perspective since the patient presented with a large mediastinal mass and no fresh tissue was available for karyotyping or flow cytometry. The final classification was rendered only after next generation sequencing (NGS) was performed. #429 was unusual due to previously unreported megakaryoblastic/erythroblastic differentiation in a *PICALM*::*MLLT10* AML. As with *BCL11B* alterations, *PICALM*fusions may show lineage ambiguity across stem cell (undifferentiated), myeloid, T-cell and mixed immunophenotypes [21–23].

#378 was of a new entity, AML with *NPM1*::*MLF1*, that was previously classified as AML-MDS. AML with *NPM1*::*MLF1* is one of the subtypes of AML where the WHO 5th Ed. and ICC differ regarding blast percentage for the diagnosis of AML (≥ 20% WHO 5th Ed. vs. > 10% ICC). The characteristic t(3;5)(q25;q35) rearrangement is often reported as the sole abnormality. An *NRAS* mutation, a common finding in AML with mutated *NPM1,*was also present in this case [38, 39]. The patient’s karyotype did not show trisomy 8, even though trisomy 8 is the most common additional abnormality associated with the t(3;5)(q25;q35) rearrangement.

#393 was a case of AML with leukemia cutis with a normal karyotype, FISH, and whole genome sequencing findings. A targeted myeloid sequencing panel revealed a *UBTF*tandem duplication (UBTF-TD). UBTF is a transcription factor that regulates ribosomal RNA expression by recruiting polymerase I (PolI) to ribosomal DNA [19, 20]. Duplications of UBTF lead to dysregulation of *HOX* gene expression, contributing to abnormal myeloid development and the characteristic myelodysplasia-related features seen in this disease. Cases with UBTF-TD AML typically have normal karyotypes or show only trisomy 8 and often have co-mutations of *WT1* or *FLT3*-ITD. Clinically, this entity is associated with poor prognosis, limited response to targeted therapies such as menin inhibitors [19].

**Fig. 5:**
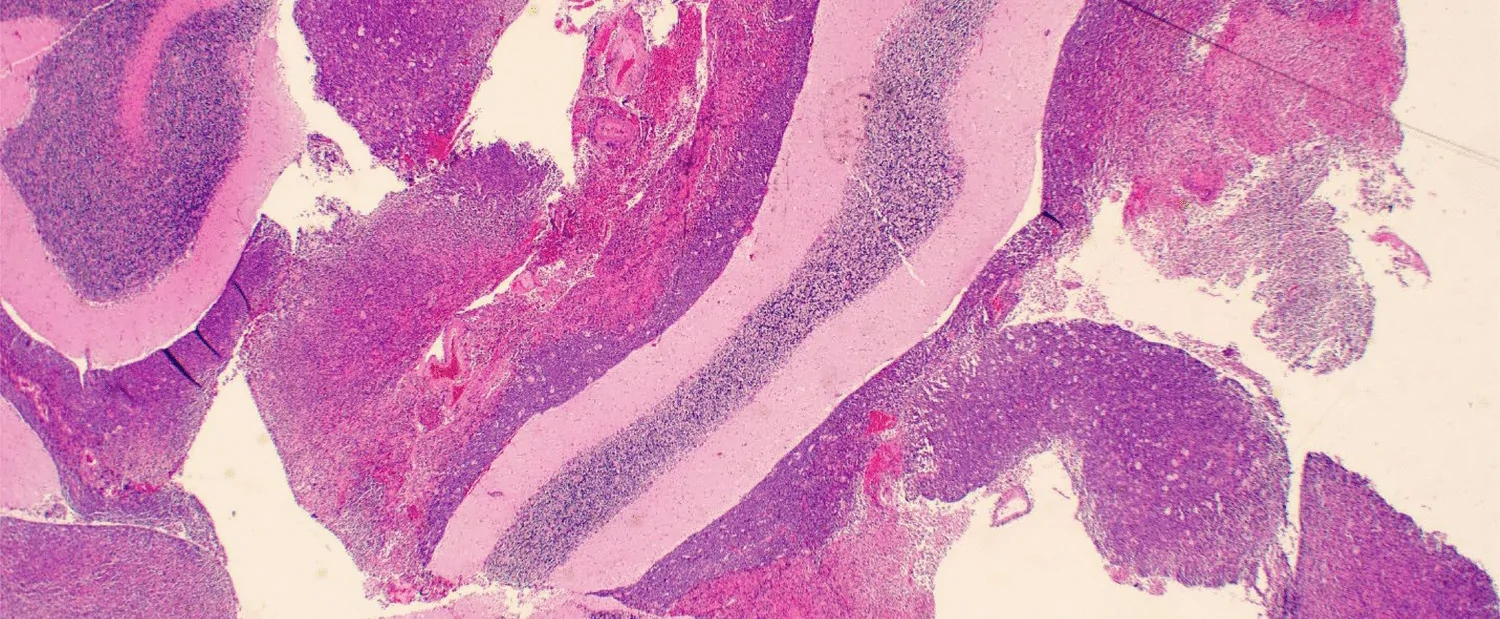
Cerebellar tissue involved by myeloid sarcoma with *KMT2A* rearrangement. Case #264, Hematoxylin and eosin (H&E). Images courtesy of submitted workshop slides, Rachel A. Mariani and colleagues, Phoenix Children’s Hospital, Phoenix, AZ

AML and MS with *KMT2A* rearrangements were the most common subtype submitted (Table 3). Case 176 harbored a *KMT2A* rearrangement and a *TP53* mutation with an MS at relapse. This would be classified as AML with *KMT2A* rearrangement in the WHO-5 classification but AML with mutated *TP53* in the ICC (Table 5). The submitters raised the possibility that nomenclature may prevent enrollment in treatment protocols involving menin inhibitors. #264 highlights several noteworthy features of MS, emphasizing that skin and lymph nodes are the most common sites of involvement, while central nervous system (CNS) disease occurs in only about 2% of presentations (Fig. 5). Because routine screening for extramedullary disease is not recommended in AML, the incidence of involvement at other anatomic sites is likely underestimated [40].

**Table 5:** Submitted cases of acute myeloid leukemia and myeloid sarcoma with *KMT2A* rearrangement

Case Number: First Author, Institution	Age (years)/Sex	Proposed Diagnosis	Panel Diagnosis	Comments
176: Zimmerman, University of Cincinnati	20/M	WHO 5th Ed: AML with *KMT2A* rearrangement (per ICC: AML with mutated *TP53*	Agree	*KMT2A* rearrangement and concurrent *TP53* mutation leading to differences in classification nomenclature; Early relapse as myeloid sarcoma
178: Haque, Yale	12/M	AML with *KMT2A* rearrangement	Agree	Two distinct blast populations by morphology and immunophenotype
264: Mariani, Phoenix Children’s	2/F	Myeloid sarcoma with *KMT2A* rearrangement	Agree	Isolated CNS presentation
395: Shi, Penn State	2/M	AML with *KMT2A*::*MLLT3* fusion and testicular myeloid sarcoma	Agree	Testicular myeloid sarcoma; CNS relapse and then marrow relapse
463: Peeples, Rutgers	16/M	AML with *KMT2A*::*MLLT3* fusion	Agree	Myeloid sarcoma; Possible *RB1* and *TP53* germline mutations

*AML* Acute myeloid leukemia, *CNS* Central nervous system, *ICC* International Consensus Classification [6], *IHC* Immunohistochemistry, *NGS* Next-generation sequencing, *WHO*World Health Organization, 5th Ed [5, 7]

Several cases fell into the categories of MDS/AML and therapy-related AML/MS and presented classification challenges (Table 6). #315 is a case that was difficult to classify because the patient’s extensive marrow fibrosis complicated sample collection and created diagnostic overlap among entities such as megakaryoblastic leukemia, MDS with fibrosis, and primary myelofibrosis. Ultimately, the findings were most consistent with MDS with fibrosis, a diagnosis that is uncommon in children and young adults.

**Table 6:** Submitted cases of myelodysplastic syndrome and therapy-related acute myeloid leukemia

Case Number: First Author, Institution	Age (years)/Sex	Proposed Diagnosis	Panel Diagnosis	
315: Bohling, Seattle Children’s	16/F	Myelodysplastic neoplasm with low blasts with progression to MDS with increased blasts/Myeloid sarcoma with megakaryocytic differentiation	Agree	Challenging to classify
306: Balakrishna, Roswell Park	35/M	AML (post cytotoxic therapy vs. inherited tumor syndrome vs. myelodysplasia-related)	AML, therapy related	Challenging to classify; Rothmund-Thompson syndrome, history of cytotoxic chemotherapy
347: Tizro, City of Hope	23/M	AML with *KMT2A* rearrangement and myelodysplasia-related cytogenetic abnormality	Agree	Challenging classification due to del(5) and *KMT2A* rearrangement
404: Venkateswaran, Memorial Sloan Kettering	21/M	AML with *KAT6A*::*CREBBP* fusion, therapy-related	Agree	Rare new entity
443: Gichinga, Beth Israel Deaconess	23/F	Donor-derived myeloid neoplasm with *GATA1* and *FANCA* variants	Agree	Sickle cell disease s/p cord blood transplant 100% donor chimerism
490: Sarra, Tufts Medical Center	26/F	AML with complex karyotype, *TP53* mutated, therapy related (ICC) Acute Myeloid Leukemia post cytotoxic therapy (MN-pCT) (WHO 5th Ed.)	Agree	Sickle cell disease s/p stem cell transplant

*AML* Acute myeloid leukemia, *MDS* Myelodysplastic syndrome

Case #347 was particularly challenging because of the co-occurrence of a rare *KMT2A* fusion partner with del(5q). The patient, a 23-year-old male, was initially classified at an outside facility as acute leukemia of ambiguous lineage favoring acute undifferentiated leukemia, based on blasts expressing CD7, CD13, CD34, CD38, and CD45 only. On re-evaluation, two distinct blast populations were apparent by flow cytometry, with CD13, CD34, and CD123 expressed uniformly; a small subset showed cytoplasmic CD3 at or above 50% of normal T-cell intensity, meeting WHO 5th Ed. criteria for T-lineage assignment but not the more stringent ICC threshold. Karyotype was near-tetraploid with del(5)(q31q35); FISH showed loss of *EGR1* at 5q31.2 and deletion of 3′*KMT2A*. NGS returned a *KMT2A*::*CBL* fusion, not currently enumerated among the ICC’s recognized *KMT2A* partners, along with *JAK3* and *MSH3* mutations and copy number loss of *CBFB* and *CBL*. The del(5q) alone would place this case in the myelodysplasia-related cytogenetic category under either WHO 5th Ed. or ICC. However, the cCD3 finding—if accepted at WHO thresholds—raises the alternative of MPAL with *KMT2A* rearrangement (T/Myeloid), an exceedingly rare designation. The panel settled on AML with *KMT2A* rearrangement and myelodysplasia-related cytogenetic abnormality, though the case makes clear that unrecognized fusion partners and inter-system differences in lineage assignment thresholds can each, independently, put a correct classification out of reach—with potential consequences for treatment protocol eligibility.

#404 describes an AML with t(8;16) *KAT6A*::*CREBBP* fusion, therapy-related, a rare newly classified entity. AML with t(8;16) *KAT6A*::*CREBBP* fusion, is recognized in both the WHO 5th Ed. and ICC and is notable for sometimes presenting with disseminated intravascular coagulation (DIC). These leukemias often show myelomonocytic differentiation, occur in about 0.5% of pediatric AML cases, and peak in the neonatal period. Congenital cases may be associated with skin lesions, which can undergo spontaneous regression. Their gene expression profile resembles that of *KMT2A*‑rearranged leukemias. Many, but not all, patients have a history of malignancy. It should be noted that adult patients with no history of malignancy and chemotherapy and a non-complex karyotype have superior outcomes [41].

Two cases (#443, 490) occurred in sickle cell patients that had undergone stem cell transplants. Case 443 described a donor-derived myeloid neoplasm after umbilical cord blood transplantation with *GATA1* and *FANCA* variants seen in germline genetic leukemia predisposition syndromes recognized in WHO 5th Ed. The presence of these variants in the setting of 100% donor chimerism suggests an underlying germline predisposition in the donor, rather than the recipient. This case underscores the clinical and ethical complexities of donor screening in umbilical cord blood transplantation, including the responsibilities related to identifying and communicating potential donor-derived genetic risks.

Three cases of MPAL were submitted to this workshop, one each of B/Myeloid, T/Myeloid, and B/T/Myeloid (trilineage) leukemia in pediatric patients ranging from 5 to 13 years of age. Collectively, the cases highlight the expanding molecular landscape of MPAL, the diagnostic challenges posed by immunophenotypic lineage overlap, and two emerging themes: *ZNF384*-rearranged MPAL as a genetically defined entity, and trilineage relapse from an initial T/Myeloid MPAL diagnosis (Table 7).

**Table 7:** WHO and ICC classifications of MPAL and associated workshop cases

WHO 5th Ed	ICC	Workshop case(s) [Number: first author, institution]
MPAL with *BCR::ABL1*	MPAL with t(9;22)(q34.1;q11.2)/*BCR::ABL1*	
MPAL with *KMT2A*	MPAL with t(v;11q23.3)/*KMT2A*	
MPAL, B/myeloid, NOS	MPAL, B/myeloid, NOS	236: Kavus H, Geisinger Medical Center
MPAL, T/myeloid, NOS	MPAL, T/myeloid, NOS	297: Blain S, HematoLogics
		471: [submitter], St. Jude Children’s Research Hospital
MPAL, NOS (rare types)	MPAL, NOS (rare types incl. B/T)	
	Provisional: ZNF384-rearranged MPAL	236: Kavus H, Geisinger Medical Center

*ICC* International Consensus Classification [6], *MPAL* Mixed phenotype acute leukemia, *NOS* Not otherwise specified, *WHO*World Health Organization, 5th Ed [5, 7]

## Mixed phenotype acute leukemia

### ZNF384-rearranged B/Myeloid MPAL

Case #236 illustrates *ZNF384*-rearranged B/Myeloid MPAL in a 13-year-old male who presented with fatigue, poor appetite, and leukocytosis (WBC 70.37 K/uL) with 32% circulating blasts. Bone marrow biopsy exceeded 90%, with complete replacement by large blasts showing fine chromatin and variably distinct nucleoli; a small subset (~ 10%) had faint cytoplasmic granularity. Flow cytometry identified blasts that were positive for CD45 (dim), CD34, CD38, CD13, CD19, HLA-DR, CD22 (dim), TdT (dim), cCD79a, and cMPO (subset), and negative for CD117, CD14, CD64, and cCD3, most consistent with a B/Myeloid immunophenotype. Karyotype showed 46,XY,del(9)(q22) in 18/20 metaphases. Next-generation sequencing (NGS) identified a *TCF3*::*ZNF384* fusion confirmed by Sanger sequencing, together with an *NRAS* mutation. The final diagnoses under both WHO-HAEM5 and ICC were MPAL, B/Myeloid with *ZNF384* rearrangement (Table 8).

**Table 8:** Submitted cases of mixed phenotype acute leukemia

Case Number: First Author, Institution	Age (years)/Sex	Proposed Diagnosis	Panel Diagnosis	Comments
236: Kavus H, Geisinger Medical Center	13/M	MPAL, B/Myeloid with *ZNF384* rearrangement (*TCF3::ZNF384*)	Agree	ZNF384-rearranged MPAL; TCF3::ZNF384 fusion by NGS; co-occurring NRAS mutation; del(9)(q22)
297: Blain S, HematoLogics/Prinses Maxima Centrum	5/M	MPAL, B/T (bilineal)	Agree	EuroFlow ALOT panel; two distinct blast populations (T-cell and B-cell); CNS involvement; MRD tracking with standardized panels
471: [Mead P], St. Jude Children’s Research Hospital	8/F	T/Myeloid MPAL at diagnosis; B/T/Myeloid (trilineage) MPAL at relapse	MPAL T/Myeloid at diagnosis; MPAL T/Myeloid at relapse	Acquisition of B-cell markers at relapse (Day 411) insufficient for B-cell lineage assignment; near-tetraploid karyotype; mutations in DNM2, EZH2, NOTCH1, NRAS, TP53; confirmed by 38-color spectral flow (SJIP38v2)

*ICC* International Consensus Classification, *MPAL* Mixed phenotype acute leukemia, *NGS* Next-generation sequencing, *WHO* World Health Organization, 5th Ed

The B/Myeloid immunophenotype and the co-occurrence of NRAS mutations seen in this case are well-recognized features of *ZNF384*-rearranged MPAL. *ZNF384* fusions account for nearly half of B/Myeloid MPAL and approximately 20% of all MPAL, with *TCF3*, *EP300*, *TAF15*, and *CREBBP* as the most common fusion partners. The transcriptional profile of *ZNF384*-rearranged B/Myeloid MPAL closely resembles its B-ALL counterpart, supporting the concept of a biological continuum, and both the WHO 5th Ed. and ICC now classify this as a genetically defined entity. The significance of del(9)(q22) in this context is uncertain.

### T/Myeloid MPAL with complex immunophenotype

Case #297 illustrates the diagnostic complexity and possible multilineage plasticity of MPAL in a 5-year-old male who presented with abdominal pain, vomiting, and weight loss. CBC showed mild anemia (hemoglobin 9.3 g/dL), normal white cell count (4.1 × 10^9^/L), and 13% circulating blasts. Bone marrow aspirate showed 83% small to medium-sized blasts with open chromatin, and a subset showing erythrocyte rosetting. CNS involvement was present. Multiparametric flow cytometry using the EuroFlow ALOT panel identified two distinct blast populations: a T-cell blast population expressing cytoplasmic CD3, CD7, CD34, CD1a (partial), CD2, CD5 (partial), CD13, and CD117 (variable) and a B-cell blast population expressing CD19, CD20 (variable), CD22, CD34, TdT, and cytoplasmic MPO. The combined immunophenotypic findings suggested bilineal B/T MPAL, with the cytoplasmic MPO (reported as partial, 60%, expression), raising consideration for an emerging myeloid component, i.e. trilineage Myeloid/B/T MPAL. However, the degree of MPO expression was challenging to estimate from the submitted flow cytometry dot plots in the context of current WHO recommendations for at least 50% expression intensity of the maximal expression control population, e.g. neutrophils [42]. Nevertheless, the case demonstrated the practical application of standardized EuroFlow panels across multiple tube configurations for resolving complex MPAL immunophenotypes and facilitating MRD tracking. There was no genetic driver identified, with subclonal *NRAS*, *NOTCH1*, and *WT1* mutations detected.

### T/Myeloid MPAL relapsing with some B-cell antigens: a diagnostic pitfall

Case #471 is of an 8-year-old female who was diagnosed with T/Myeloid MPAL based on flow cytometry showing 16% blasts positive for CD34, HLA-DR, CD33, CD11b, CD7, cytoplasmic CD3, CD38, CD45, CD64, CD117, CD11c, CD13 (dim), CD56 (subset), and MPO, without B-lineage markers. At Day 411 relapse, bone marrow showed 46% blasts that retained the T/Myeloid phenotype but had newly acquired dim CD19 and cytoplasmic CD79a expression. This acquisition of B-cell markers was confirmed by both conventional 8-color flow cytometry and a 38-color spectral immunophenotyping panel (SJIP38v2), with MPO positivity in 3–5% of blasts by cytochemistry in agreement. Cytogenetics at relapse revealed a near-tetraploid complex karyotype (~ 83 chromosomes). NGS identified mutations in *DNM2*, *EZH2*, *NOTCH1*, *NRAS*, and *TP53*genes. While the submitters raised consideration for tri-lineage commitment (MPAL B/T/Myeloid), B-cell antigen expression, particularly CD79a expression, is relatively common in T-ALL [43]. Moreover, in the absence of strong CD19 expression (i.e. weak or dim CD19 as in this case), current WHO and ICC definitions of B-cell lineage commitment require at least two additional B-cell markers to handle just such examples [5, 6]. The panelists reclassified this case as relapsed T-ALL with immunophenotypic and genetic evidence of clonal evolution.

## Miscellaneous entities

Four cases in this session did not fit cleanly into established MPAL or AML categories and collectively raise important questions about entities that may warrant formal recognition in future classification schemes. Three cases featured biallelic loss of *SMARCB1* (*INI1*/*BAF47*), and one case illustrated the diagnostic pitfalls of blastic plasmacytoid dendritic cell neoplasm (BPDCN) presenting in a lymph node of a child.

### SMARCB1-deficient hematopoietic malignancies

**Fig. 6:**
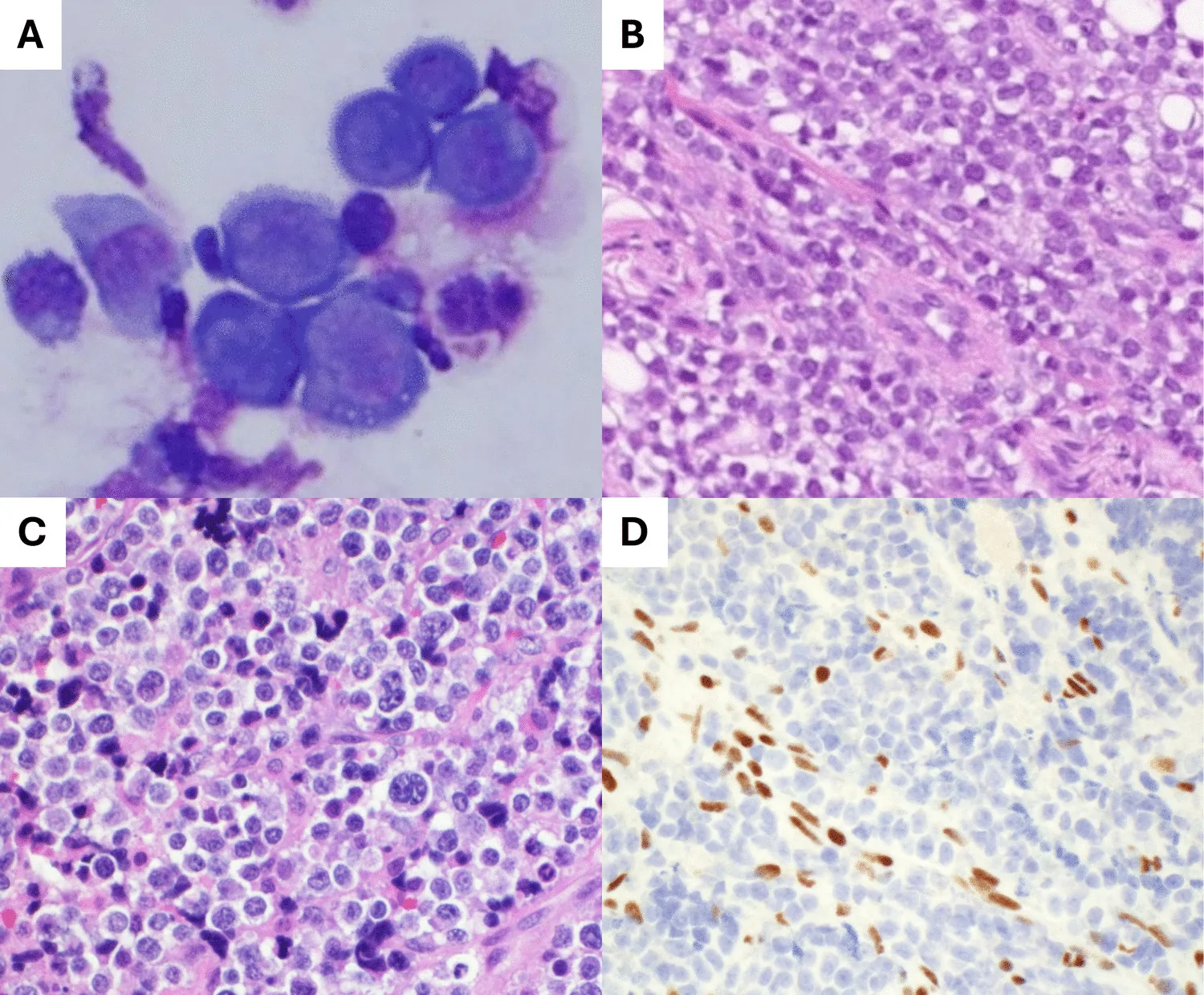
*SMARCB1*-deficient hematopoietic malignancies. **A** Case #292, cerebrospinal fluid cytology from a 12-year-old male. Diff-Quik. Image courtesy of submitted workshop slides, Sadhika Sood and colleagues, New York University Grossman School of Medicine, New York, NY. **B** Case #312, skin biopsy from 4-year-old female. H&E. Image courtesy of submitted workshop slides, Nadia Nasir and colleagues, National Cancer Institute, National Institutes of Health, Bethesda, MD. **C** H&E and D. INI1 immunostain from Case #354, lymph node biopsy from a 14-year-old male. Image courtesy of submitted workshop slides, Mariko Yabe and colleagues, Memorial Sloan Kettering Cancer Center

Three cases of aggressive hematopoietic malignancy with biallelic *SMARCB1* loss were submitted to this workshop, arriving from multiple institutions, yet sharing a strikingly coherent set of features that collectively support recognition of this entity as a discrete category (Fig. 6).

Case #292 described a 12-year-old male who developed a nine-month history of daily headaches, photophobia, progressive ataxia, and 30-pound weight loss. Initial brain biopsies showed only mixed inflammatory infiltrate with eosinophilia, and empiric steroids and IVIG failed. On re-presentation, CSF analysis revealed markedly elevated cytokines, elevated protein, and 33% myeloid precursors by flow cytometry. Brain and dural biopsies showed atypical immature mononuclear cells with pleomorphism infiltrating the parenchyma; CSF cytology showed immature cells with fine chromatin and prominent nucleoli. Immunophenotyping by flow cytometry and IHC showed CD45, CD33, CD117, CD38, CD7, CD43, cytoplasmic CD3 (weak), CD34, and PD-L1 positivity, with loss of INI-1 by IHC, and negativity for CD5, CD123, CD14, CD2, MPO, and MUM1. The patient was treated with intrathecal chemotherapy without significant improvement and died of progressive disease. This case represents an extremely rare presentation of SMARCB1-deficient hematopoietic malignancy arising exclusively in the CNS leptomeninges.

Case #312 described a 4-year-old female with cervical lymphadenopathy that partially responded to antibiotics. Excisional biopsy showed near-total nodal infarction; needle core biopsy revealed a diffuse proliferation of medium to large pleomorphic lymphoid cells with prominent nucleoli and abundant eosinophilic cytoplasm. Skin biopsy obtained three months later showed an atypical lymphoid infiltrate dissecting dermal collagen with perivascular and periadnexal distribution. IHC showed positivity for CD2 (focal, weak), CD5, CD7, CD43, CD33 (focal), and loss of INI-1 expression, with negativity for CD3, MPO, CD117, TdT, CD1a, and CD34; TCR gamma gene rearrangement was absent by PCR. NGS demonstrated *SMARCB1* loss, *CDKN2A* frameshift, and *BARD1* frameshift. The patient experienced progressive disease through CHOEP chemotherapy before proceeding to bone marrow transplantation and achieving remission.

Case #354 described a 14-year-old male with autism spectrum disorder and growth delay who developed progressive dyspnea and left shoulder pain due to a large heterogeneous mediastinal mass with bilateral supraclavicular and cervical lymphadenopathy. Biopsy showed effacement by a diffuse atypical mononuclear proliferation with irregular nuclear contours and vesicular chromatin, scattered pleomorphic cells with occasional multinucleation, numerous mitoses including atypical forms, and focal necrosis. IHC showed expression of CD45, CD2, CD4 (subset), CD7, CD13, CD43 (subset), CD79a, and MPO (dim), with loss of INI-1, and negativity for CD3, CD5, CD20, CD30, CD34, CD56, CD117, TdT, PAX5, TCR beta, TCR delta, and ALK1. Flow cytometry showed 86% viable cells with an inconclusive T-predominant phenotype. Complex karyotype was present. NGS identified *TP53* p.D281H (VAF 4%) and co-deletion of *BCR* and *SMARCB1* at 22q11.23 (fold change − 11.5), without additional candidate drivers on whole-exome sequencing. RNA-Seq was negative for gene fusions. DNA methylation profiling placed the tumor within the acute leukemia cluster supporting its classification as a hematologic malignancy. The patient received DA-EPOCH with initial 68% reduction in mass size but progressed after six cycles; salvage with brentuximab vedotin, nivolumab, and bendamustine failed, and the patient died within 6 months of diagnosis. The proposed diagnosis was aggressive hematopoietic neoplasm with biallelic *SMARCB1* loss presenting with myeloid, B-cell, and T/NK-cell differentiation. In both this and the previous case, INI-1 loss by IHC implies biallelic *SMARCB1* loss, which could not be definitely confirmed by the standard NGS panels in either submitted case.

Considered together, these three cases define a clinicopathologic syndrome of *SMARCB1*-deficient hematopoietic malignancy with the following unifying features: young patient age (4–14 years); a partially aberrant T-cell immunophenotype with variable myeloid marker co-expression and frequent loss or weak expression of pan-T markers; absence of clonal TCR rearrangement in most cases; biallelic *SMARCB1* deletion as the primary oncogenic event (in some cases with co-deletion of neighboring 22q11 genes including BCR); and an extremely aggressive clinical course with poor response to conventional lymphoma or leukemia regimens. Methylation profiling in case #354 positioned the tumor within the acute leukemia cluster, providing an important molecular anchor. While the WHO 5th Ed. and ICC do not currently include a formal category for *SMARCB1*-deficient hematopoietic malignancy, recent literature has described this entity as a subset of aggressive T-cell lymphoma [44] or acute leukemia of ambiguous lineage [45, 46]. The absence of a consensus name and the rarity of the entity present significant diagnostic and therapeutic challenges; clinical testing for SMARCB1 by IHC (INI-1 loss) or NGS should be incorporated into the workup of any aggressive T-lineage or lineage-ambiguous hematopoietic neoplasm in young patients, particularly when conventional lymphoma or leukemia-associated mutations are absent.

### Blastic plasmacytoid dendritic cell neoplasm presenting as a lymph node mass

Case #60 illustrates the diagnostic challenge posed by BPDCN when presenting in an extracutaneous site without classic clinical features. A 9-year-old male presented with a 2-week history of an enlarging, painful right upper thigh mass. CBC was normal. Imaging showed two right inguinal lymph node lesions, initially treated as reactive with two courses of antibiotics without response, prompting excisional biopsy. Histology showed a diffuse infiltrate of small monomorphic cells with fine chromatin, indistinct nucleoli, and scant cytoplasm, with frequent mitoses. Flow cytometry demonstrated dim CD45, HLA-DR, CD33 (dim), CD2 (heterogeneous), CD4, CD5, CD7 (heterogeneous), and negativity for CD34, cytoplasmic CD3, CD1a, CD8, MPO, CD11b, CD13, CD14, CD15, CD64, CD117, CD19, PAX5, cytoplasmic CD22, and cytoplasmic CD79a. Surface CD3 was present on a very small subset, and TdT was positive, leading to initial classification as T-lymphoblastic leukemia/lymphoma. However, molecular studies revealed *ASXL1* p.S965* (VAF 40.6%), RB1 copy number loss (13q), and a *MYB::PLEKHO1* chromosomal rearrangement by RNA-Seq. Recognition of the *MYB*rearrangement, which has been reported in BPDCN, particularly in pediatric patients [47–49], prompted additional IHC, which confirmed CD4, CD56, CD123, and TCL1 positivity, establishing the diagnosis of BPDCN.

This case highlights two important diagnostic points. First, BPDCN can present in lymph nodes in children, mimicking T-lymphoblastic leukemia/lymphoma by morphology, flow cytometry, and TdT positivity. The absence of MPO and B-lineage markers, combined with the T-cell marker pattern (CD4+, CD5+, CD7+, with only a small surface CD3-positive fraction), should prompt inclusion of CD56 and CD123 in the diagnostic workup when morphologic features and the full immunophenotypic picture do not fit a typical T-ALL pattern. Second, recurrent *MYB* rearrangements, including *MYB::PLEKHO1* and other *MYB* fusions, are emerging as genomic signatures of BPDCN enriched in pediatric cases and should be recognized as diagnostically informative by molecular pathologists interpreting RNA-Seq or NGS results in this context.

## Data Availability

No datasets were generated or analysed during the current study.
